# Colonic perineurioma (benign fibroblastic polyp): case report and review of the literature

**DOI:** 10.1186/s13000-018-0694-z

**Published:** 2018-02-20

**Authors:** Abraham Christoffel van Wyk, Hennie van Zyl, Jonathan Rigby

**Affiliations:** 1 Division of Anatomical Pathology, National Health Laboratory Service, Faculty of Medicine and Health Sciences, Tygerberg Hospital, Stellenbosch University, Cape Town, South Africa; 2 Department of Surgery, Karl Bremer Hospital, Cape Town, South Africa

**Keywords:** Colonic perineurioma, Fibroblastic polyp, BRAF mutation, Hemosiderin

## Abstract

**Background:**

Colorectal perineuriomas are uncommon benign mucosal-based proliferations of mesenchymal cells that express perineurial markers, often associated with colonic crypts displaying a serrated/hyperplastic architecture. The vast majority of cases arise distal to the splenic flexure and have been described as sessile polyps. Using molecular analysis, BRAF mutations have been demonstrated in the serrated crypt epithelium. We report a new case of perineurioma presenting as a pedunculated polyp in the transverse colon, with prominent hemosiderin deposits in the uninvolved lamina propria that separated the perineurial proliferation from the surface epithelium, a previously unreported histological finding. By using immunohistochemistry, we demonstrated the presence of BRAF V600E mutated protein in the serrated crypt epithelium. In addition, a review of the literature on colorectal perineurioma is provided.

**Case presentation:**

A 5 mm pedunculated polyp was removed from the transverse colon of a 42 year old man who presented with epigastric pain, weight loss and rectal bleeding. A proliferation of uniform plump spindled cells expanded the lamina propria and separated serrated colonic crypts. The epithelial component closely resembled microvesicular hyperplastic polyp. Immunohistochemical stains for epithelial membrane antigen (EMA), glucose transporter 1 (GLUT1) and collagen IV were positive in the stromal proliferation. A mutation-specific monoclonal antibody directed against BRAF V600E showed positive cytoplasmic staining in the serrated crypt epithelium but not in the perineurial proliferation. Conspicuous hemosiderin deposition was seen in the inflamed lamina propria between the perineurial proliferation and the surface epithelium.

**Conclusion:**

Although the majority of colorectal perineuriomas occur in the sigmoid colon and rectum and are described as sessile polyps, colorectal perineurioma can present as a pedunculated polyp proximal to the splenic flexure as described in this case. Conspicuous hemosiderin deposition can be seen in the superficial lamina propria. BRAF mutations are limited to the serrated crypt epithelium.

## Background

Colorectal perineuriomas, first described as benign fibroblastic polyps in 2004 by Eslami-Varzaneh et al., [1] are benign mucosal-based mesenchymal polyps characterized by a proliferation of benign stromal spindled cells expressing perineurial markers. The perineurial proliferation is often associated with serrated colonic crypts resembling a microvesicular hyperplastic polyp [2]. BRAF mutations have been demonstrated in the serrated crypt epithelium but not in the perineurial proliferation [3–6]. The vast majority of colorectal perineuriomas occur distal to the splenic flexure [5] and are described as sessile [7–9]. We report the clinical, histological and immunohistochemical features of a new case of colonic perineurioma and provide a review of the literature.

## Case presentation

A 42 year old man with a history of epigastric pain for two years, weight loss and an episode of rectal bleeding was referred for colonoscopy after gastroscopy revealed only antral gastritis. Physical examination was unremarkable except for the presence of mild epigastric tenderness. Serum lipase levels, liver function tests and full blood count were essentially normal. A pedunculated polyp, 5 mm in diameter, was removed from the transverse colon and sent for histopathological evaluation. No other endoscopic abnormalities were identified.

**Fig. 1:**
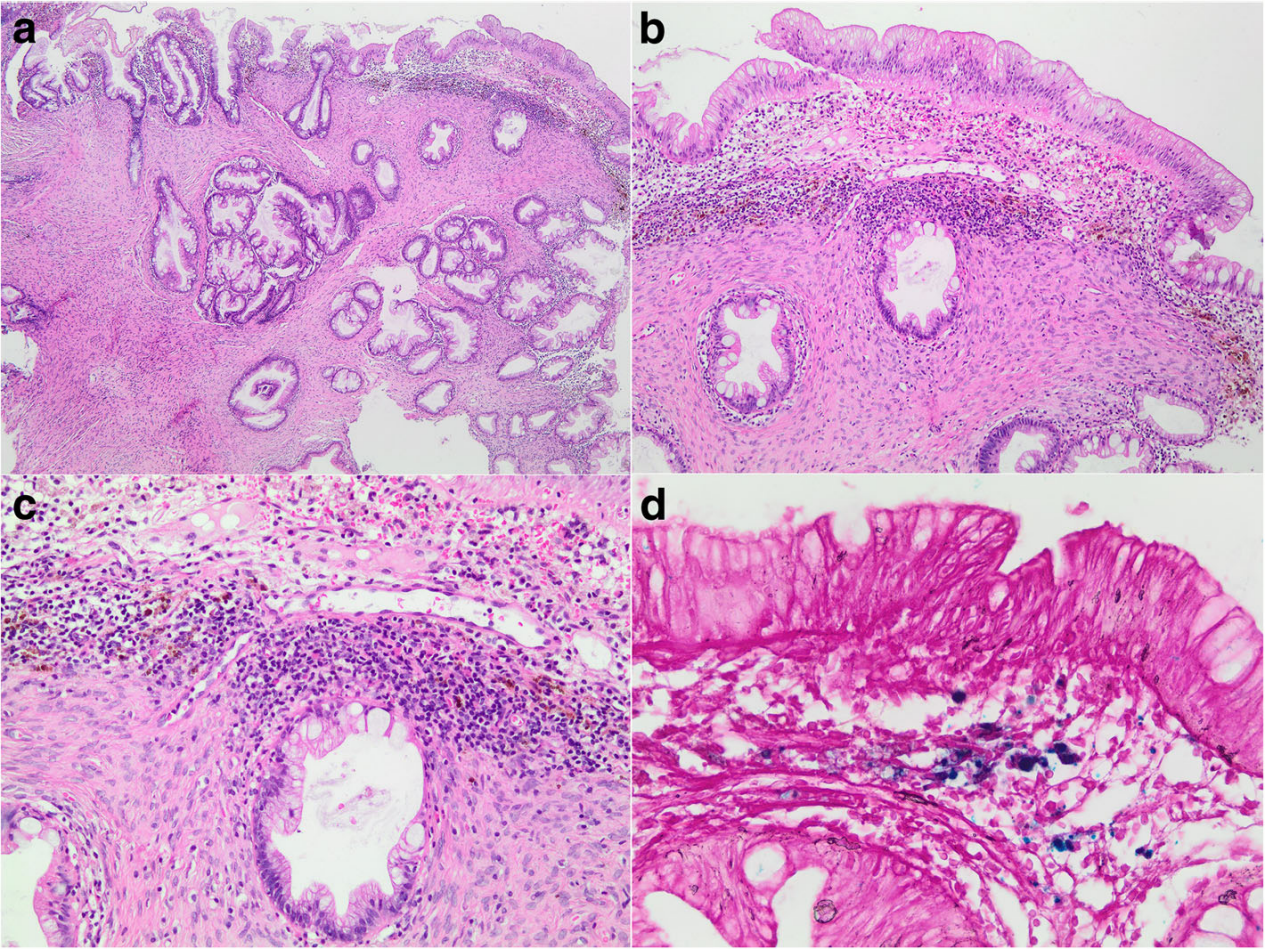
Histological findings. **a** A proliferation of benign-appearing eosinophilic spindled cells expanded the lamina propria and separated colonic crypts with serrated architecture (haematoxylin and eosin, original magnification 40×). **b** The proliferation of spindled cells was separated from the surface epithelium by a zone of uninvolved but inflamed lamina propria (haematoxylin and eosin, original magnification 100×). **c** Conspicuous hemosiderin deposition occurred at the interface of the spindle cell proliferation and the inflamed lamina propria (haematoxylin and eosin, original magnification 200×). **d** A Perls’ Prussian blue stain confirmed the presence of hemosiderin (original magnification 400×)

On histological examination, a proliferation of uniform, plump spindled cells in the lamina propria surrounded and separated serrated and nonserrated crypts that resembled those of microvesicular hyperplastic polyps (Fig. 1a and b). A separate fragment showed morphological features identical to those of a hyperplastic polyp and did not show the spindled stromal proliferation seen in the first fragment. The spindled cells had eosinophilic cytoplasm, indistinct cell borders and ovoid to spindled nuclei with inconspicuous nucleoli. Necrosis, pleomorphism and mitotic figures were absent. The surface epithelium was separated from the stromal proliferation by a zone of uninvolved but inflamed lamina propria containing ectatic capillary vessels. Inflammatory cells in this zone included lymphocytes, frequent eosinophils, occasional histiocytes and a few plasma cells. Prominent hemosiderin deposition, confirmed with Perls’ prussian blue stain, occurred at the interface of the stromal proliferation and the superficial lamina propria (Fig. 1c and d). Extravasated red blood cells occurred in the superficial lamina propria just below the surface epithelium.

**Fig. 2:**
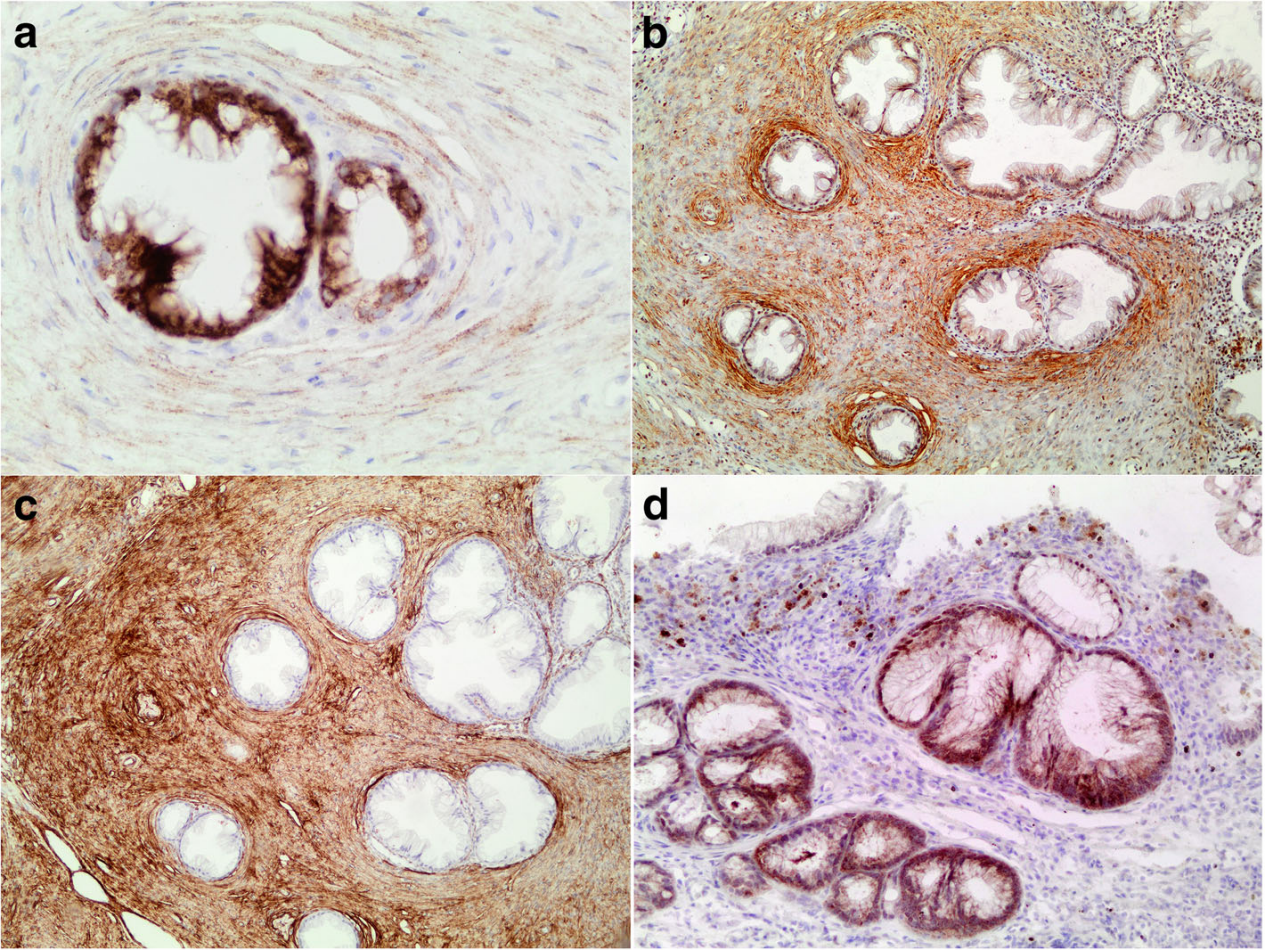
Immunohistochemical findings. **a** Staining for EMA showed weak delicate staining in the stromal component compared to strong staining in the serrated crypts (original magnification 400×). **b** GLUT1 showed positive staining with pericryptic accentuation (original magnification 100×). **c** Moderate to strong staining was seen with collagen IV (original magnification 100×). **d** Staining for BRAF V600E showed cytoplasmic expression in serrated crypt epithelium but was negative in nonserrated crypts (nuclear staining only; uppermost crypt) and in the perineurial proliferation. The pigment in the lamina propria is hemosiderin (original magnification 200×)

Immunohistochemical staining with a monoclonal epithelial membrane antigen (EMA) antibody, using clone E29 (DAKO, Glostrup, Denmark) at an antibody concentration of 1:500, showed weak positive staining in the stromal proliferation (Fig. 2a) while both glucose transporter 1 (GLUT1) (Fig. 2b) and collagen type IV (Fig. 2c) showed moderate to strong staining. GLUT1 expression was accentuated around crypts. Stains for CD34, CD117, desmin, α-smooth muscle actin and S100 protein were all negative. Desmin highlighted a disorganized muscularis mucosae with a few smooth muscle cells that extended into the perineurial proliferation. A monoclonal antibody directed against the mutated protein BRAF V600E (clone VE1, Ventana Medical Systems) showed moderately intense cytoplasmic staining in serrated colonic crypt epithelium but was negative in nonserrated crypt epithelium (nuclear staining only) and in the perineurial proliferation (Fig. 2d).

## Discussion

### Historical perspective

Colorectal perineuriomas, although uncommon, are probably underrecognized lesions [8]. These benign mesenchymal polyps were first described as benign fibroblastic polyps of the colon in a series of 14 cases by Eslami-Varzaneh et al. in 2004 [1]. In the following year, Hornick and Fletcher reported 10 cases of intestinal perineuriomas and acknowledged the morphological similarities with the then recently described benign fibroblastic polyp [8]. In 2006, Zamecnik and Chlumska [10] reported expression of perineurial immunohistochemical markers in cases that they had previously reported as benign fibroblastic polyps [11]. They suggested that many, if not all fibroblastic polyps, should be reclassified as perineuriomas. This notion was supported by Groisman and Polak-Charcon [12] after studying a series of 28 cases (of which 10 were previously reported as fibroblastic polyps [2]) in which immunohistochemical markers of perineurial differentiation were expressed. They emphasized that EMA expression may be extremely limited or faint and that at least 2 markers of perineurial differentiation should be used to reach an accurate diagnosis.

### Clinical demographics

Using a PubMed search and reference lists of published articles on colorectal perineurioma / fibroblastic polyp, a total of 157 reported cases were found [1–5, 7–17]. A further 38 cases appeared as published congress abstracts [6, 18]. The clinical features of previously reported colorectal perineuriomas, including the current case, are summarized in Table 1.

**Table 1:** Clinical features of previously reported colorectal perineuriomas including the current case (Total: 158)

Ref.	No of cases	Mean age in years (Range)	F:M	Distal to transverse colon	Mean size (Range) in mm	Endoscopic description	No of cases with polyps elsewhere in colon
[1]	14	62 (37-84)	8:6	13/14	5.1 (2 – 15)	‘polyps’	10/14
[10, 11]	5	65 (52-77)	3:2	4/5	3 (2 – 4)	NS	4/4
[8]	9	51 (35-72)	7:2	7/8	6.8 (2-30)	Small sessile polyps	5/9
[7]	4	66 (58-71)	2:2	3/4	9.8 (6-15)	Two sessile polyps depicted	3/4
[2, 5, 12, 15]	60	60 (36-84)	30:30	52/60	3.4 (1-8)	Flat/sessile to round/ pedunculated	21/60
[9]	4	59 (47-80)	3:1	4/4	3.8 (3-5)	Sessile polyps	3/4
[13]	1	54	0:1	NS	5	NS	NS
[14]	1	50	0:1	1/1	6	Polypoid lesion	0/1
[3]	29	64 (43-84)	23:6	26/28	2.7 (3-9)	NS	19/29
[4]	20	58 (44-87)	9:11	17/20	5.1 (3-15)	NS	15/20
[16]	9	62 (45-84)	5:4	4/9	4 (NS)	NS	6/9
[17]	1	51	1:0	1/1	15	‘Submucosal lesion’	0/1
Current case	1	42	0:1	0/1	5	Pedunculated polyp	0/1
Total	158	60 (35-87)	F:M = 1.3	132/155 85%	4.1 (1 – 30)		86/156 55%

*F* Indicates female, *M* Male, *NS* Not specified

The mean age at diagnosis for published cases was 60 years (range 35 – 87 years). A slight female predominance was noted (F:M = 1.3).

For those cases where the reason for the colonoscopy was indicated, 51 of 74 (69%) were for routine screening, 3 of 74 (4%) for screening in a patient with previous colorectal carcinoma, 10 of 74 (13%) for gastrointestinal bleeding or occult blood in the stools, 7 of 74 (9%) for abdominal pain and one each for diarrhoea, change of bowel habit and thickening of large bowel wall on computed tomography scan. Although the perineurioma might have been responsible for some of these presenting symptoms and signs, the associations might have been purely coincidental.

Almost three quarters of reported cases occurred in the sigmoid colon or rectum. Less than 15% of cases were located proximal to the splenic flexure. The average size was 4.1 mm (range: 1 – 30 mm). Most of the polyps were described as small sessile polyps [7–9] but Groisman et al. reported that the polyps could be flat/sessile or rounded/pedunculated [5, 12]. Concurrent polyps elsewhere in the gastrointestinal tract (mainly adenomas and / or hyperplastic polyps) were reported in 86 of 156 cases (55%). No recurrences or metastases have been reported.

### Microscopic findings

The typical histological appearance was that of an intramucosal proliferation of plump, uniform spindle cells that filled the lamina propria causing separation, distortion and entrapment of colonic crypts. The spindled cells had pale eosinophilic cytoplasm with indistinct cell borders within a fine collagenous stroma. The cells often had a concentric arrangement around crypts or glands. A thin zone of uninvolved, mildly inflamed lamina propria separated the perineurial proliferation from the overlying surface epithelium in many cases. The presence of hemosiderin deposition has not been specifically described in colorectal perineuriomas, although conceptually, it can occur in any pedunculated polyp. It has been described in hyperplastic polyps [19] as an indicator of previous bleeding, presumably as a result of local trauma. This finding suggests that the perineurioma could be the cause of the rectal bleeding in our patient.

Hemosiderin deposition in the lamina propria was reported as a common finding in inflammatory myoglandular polyps [20], polyps that can also be associated with serrated crypts and may be considered in the differential diagnosis of perineurioma. One of the cases reported as a benign fibroblastic polyp of the colon [14] indeed had morphological features associated with inflammatory myoglandular polyp.

Mild disorganization of the muscularis mucosae with occasional thin bundles extending towards the surface was reported [1]. No significant pleomorphism, necrosis or mitotic figures were identified. Three quarters of cases (108 of 143) were associated with serrated crypts (Table 2), most often resembling microvesicular type hyperplastic polyps. Submucosal involvement has been reported [5, 12] although the absence of submucosa in most biopsies makes it difficult to give an accurate percentage of cases with submucosal involvement. One of the cases included in this review was restricted to the submucosa [8] and may represent a conventional soft tissue perineurioma. A “lipoma-like” proliferation underlying the mucosal perineurial proliferation was seen in 6 cases [5].

**Table 2:** Histological, immunohistochemical and molecular findings in reported colorectal perineuriomas (Total: 158)

Ref.	No of cases	Serrated crypts	EMA+^a^	Claudin-1+	GLUT1+	Collagen IV+	CD34+^a^	BRAF mutation
[1]	14	3/14	0/14	ND	ND	ND	3/14	ND
[10, 11]	5	2/4	3/5	4/5	5/5	ND	2/4	ND
[8]	9	5/9	9/9	4/9	ND	ND	2/9	ND
[7]	4	4/4	ND	ND	ND	ND	0/4	ND
[2, 5, 12, 15]	60	45/60	39/45	40/45	42/45	45/45	0/10	5/20
[9]	4	NS	4/4	4/4	4/4	ND	ND	ND
[13]	1	1/1	NS	NS	NS	1/1	ND	ND
[14]	1	0/1	ND	ND	ND	ND	0/1	ND
[3]	29	29/29	21/26	15/17	20/26	17/17	8/27	14/22
[4]	20	18/20	20/20	ND	ND	ND	ND	18/20
[16]	9	NS	7/9	9/9	7/9	ND	ND	ND
[17]	1	NS	0/1	ND	1/1	1/1	1/1	ND
Current case	1	1/1	1/1	ND	1/1	1/1	0/1	1/1 (IHC)
Total	158	108/143 75%	104/134 78%	76/89 85%	80/91 88%	65/65 100%	16/71 23%	38/63 60%

^a^EMA and CD34 staining often described as weak and/or focal

*ND* Not done, *NS* Not specified, *IHC* Immunohistochemistry

### Differential diagnosis and immunohistochemical findings

The diagnosis of perineurioma can be suspected with reasonable confidence on morphology alone in typical cases with associated serrated epithelium but the demonstration of expression of at least two perineurial markers is recommended for accurate diagnosis [12]. It is prudent to exclude gastrointestinal stromal tumour (the only entity with malignant potential in the differential diagnosis) in cases with clinical or pathological suspicion of origin deep to the mucosa. Entities which may be considered in the differential diagnosis are compared in Table 3. Gastrointestinal neurofibromas are strongly associated with neurofibromatosis type 1 and patients with multiple ganglioneuromas may have Cowden syndrome which make accurate diagnosis important even among the benign entities [21].

**Table 3:** Comparison of colorectal perineurioma with entities in the differential diagnosis

	Perineurioma	GIST [25]	Schwann cell hamartoma [21]	Ganglio-neuroma [25]	Neurofibroma [21]	Schwannoma [26]	Inflammatory fibroid polyp [25]	Inflammatory myoglandular polyp [20]	Leiomyoma of the MM [27]
Most common location	Distal colon	Stomach, small bowel	Distal colon	Distal colon	Stomach, small bowel	Stomach	Stomach, ileum	Distal colon	Distal colon
Epicentre	Mucosa	MP	Mucosa	Mucosa	Submucosa	MP	Submucosa	Mucosa	MM
Typical size	< 10 mm	Two-thirds > 50 mm	≤5 mm	10 - 20 mm	Wide size range	> 10 mm	10 - 50 mm	5 - 20 mm	< 10 mm
Histological clues	Entrapped serrated crypts, peri-cryptic growth, bland cytology	Spindled to epithelioid, variable palisading, paranuclear vacuoles, collagen fibrils	Ample eosinophilic cytoplasm, no serration in entrapped crypts	Ganglion cells present	Diverse cellular composition: Schwann cells, fibroblasts, perineurial-like cells, axons	Circumscribed, peripheral lymphoid cuff with germinal centres, focal atypia	Many eosinophils, perivascular concentric cuffing, fibromyxoid background	Inflamed granulation tissue, proliferation of smooth muscle in LP, occasional cystic glands	Originates from MM, circum-scribed, eosinophilic cytoplasm
Positive IHC stains	EMA, claudin1, GLUT1	C-Kit, DOG1, CD34	S100 (all cells), NFP (rare)	S100 in spindled cells	S100 (subset of cells), CD34	S100, GFAP	CD34, cyclin D1, fascin	SMA, desmin in smooth muscle	SMA, desmin

*GIST* Gastrointestinal stromal tumour, *MM* Muscularis mucosae, *MP* Muscularis propria, *LP* Lamina propria, *EMA* Epithelial membrane antigen, *GLUT1* Glucose transporter 1, *DOG1* Discovered On GIST 1, *NFP* Neurofilament protein, *GFAP* Glial fibrillary acidic protein; *SMA* Smooth muscle actin

The immunohistochemical features of previously reported colorectal perineuriomas, including the current case, are summarized in Table 2. GLUT1 and claudin 1 showed strong and diffuse immunoreactivity in 88% and 85% of cases respectively. EMA expression, although present in 78% of cases, was often focal and weak and should be evaluated on high magnification to appreciate the delicate membranous staining pattern [8]. Some authors have proposed high antibody concentration and/or an enhanced antibody retrieval protocol to demonstrate EMA expression [12]. Collagen IV expression was demonstrated in all cases in which the stain was performed but is not specific for perineurioma. CD34 expression was seen in 23% of cases but was generally reported as limited and focal. Vimentin was expressed in all cases in which the stain was done but, due to its low specificity, is usually not diagnostically helpful. Desmin and C-Kit (CD117) were consistently negative, helping to exclude inflammatory myoglandular polyp and gastrointestinal stromal tumour respectively. S100 protein expression, although reported in one case [16], would usually raise suspicion of a benign nerve sheath tumour (Table 3). Other negative immunohistochemical stains included broad spectrum cytokeratins, h-caldesmon, CD31, BCL2, cyclin D1, CD21, CD23 and CD35. Ki-67 (MIB1) proliferation index was less than 1% in 15 cases in which the stain was performed [2, 7, 17].

The current case is the first reported colorectal perineurioma where the BRAF V600E mutated protein was demonstrated by immunohistochemical detection. Interpretation of the BRAF V600E immunohistochemical stain was however not straightforward due to moderately intense nuclear staining in lesional and non-lesional colonic epithelial cells. Difficulties in the interpretation of immunohistochemistry for BRAF in serrated lesions of the colon have been highlighted by other authors [22].

### Electron microscopy findings

Ultrastructural features of perineuriomas include parallel, elongated spindled cells with collagen-rich intervening matrix, long thin cytoplasmic processes with frequent pinocytotic vesicles and junctional complexes and a discontinuous basal lamina [23].

Eight gastrointestinal cases from four studies showed perineurial features on ultrastructural examination [8–10, 12]. This included one submucosal jejunal perineurioma [8] and three re-examined large bowel cases, initially reported as fibroblastic polyps [10, 12]. Early studies done before the relationship between fibroblastic polyps and perineuriomas became generally known, reported fibroblastic characteristics on electron microscopy [1, 7]. Ultrastructural studies were done on tissue retrieved from paraffin wax blocks which might have made identification of pinocytotic vesicles and basal lamina difficult [12].

### Molecular findings

Agaimy et al. demonstrated p.V600E *BRAF* mutations in 63% of 22 cases of perineurioma (all associated with serrated architecture) [3]. Using an allele-specific real-time polymerase chain reaction assay, Pai et al. found p.V600E mutations in 18 of 20 perineuriomas [4]. Notably, all 18 perineuriomas positive for the mutation were associated with serrated crypts while the two cases without the mutation had a perineurial stromal proliferation only without serrated epithelium. Further support for the notion that *BRAF* mutations occur only in the epithelial component and not in the stromal component came from a study by Groisman et al. who demonstrated *BRAF* mutations (using direct sequencing) in 5 of 8 serrated perineuriomas while none of 12 cases of non-serrated perineuriomas harboured a *BRAF* mutation [5]. Interestingly, while all previous studies found exclusively a p.V600E mutation, two of the mutations in this study were p.V600R mutations. The observation of positive cytoplasmic staining for a BRAF V600E immunohistochemical stain in the serrated epithelium, but not in the perineurial proliferation in our case, supports the findings of these studies.

At this stage the exact nature and origin of the perineurial proliferation is unclear. It has been suggested that the perineurial stromal component might be derived from modified pericryptic fibroblasts as a consequence of a yet poorly understood epithelial-stromal interaction [3]. Perineurial-like stromal proliferations have also been reported in sessile serrated adenomas (SSA) and other serrated polyps. Pai et al. reported perineurial-like stromal proliferations in 6.5% of 198 consecutive SSA [4]. Thompson et al. demonstrated claudin-1 positive spindle cell proliferations in 9.3% of 377 serrated polyps which included SSA, microvesicular hyperplastic polyps and serrated polyps not otherwise specified [24]. 82% of these polyps harboured *BRAF* mutations which led them to suggest that there was a strong indication of epithelial-mesenchymal interactions in BRAF positive serrated polyps and the possibility of epithelial mesenchymal transformation occurring in a proportion of serrated polyps. Further studies are necessary to assess clonality and gene alterations in the perineurial stromal component of colorectal perineurioma.

## Conclusion

In conclusion, although the majority of colorectal perineuriomas occur in the sigmoid colon and rectum and are usually reported as sessile, colorectal perineurioma can present as a pedunculated polyp proximal to the splenic flexure as demonstrated in this case. The polyp showed conspicuous hemosiderin deposition in the overlying uninvolved lamina propria and, by using immunohistochemistry, we demonstrated BRAF mutated protein restricted to the serrated crypt epithelium.

## Abbreviations

EMA: Epithelial membrane antigen
GLUT1: Glucose transporter 1
SSA: Sessile serrated adenoma
